# Machine learning to detect Alzheimer's disease with data on drugs and diagnoses

**DOI:** 10.1016/j.tjpad.2025.100115

**Published:** 2025-03-08

**Authors:** Johanna Wallensten, Caroline Wachtler, Nenad Bogdanovic, Anna Olofsson, Miia Kivipelto, Linus Jönsson, Predrag Petrovic, Axel C. Carlsson

**Affiliations:** a Department of Clinical Sciences, Danderyd Hospital, 18288, Stockholm, Sweden; b Academic Primary Health Care Centre, Region Stockholm, Sweden; c Division of Family Medicine and Primary Care, Department of Neurobiology, Care Sciences and Society, Karolinska Institutet, Alfred Nobels allé 23, 14183 Huddinge, Sweden; d Division of Clinical Geriatrics, Center for Alzheimer Research, Department of Neurobiology, Care Sciences and Society, Karolinska Institutet, 17177, Stockholm, Sweden; e Division of Biostatistics, Institute of Environmental Medicine, Karolinska Institutet, 17177, Stockholm, Sweden; f Theme Inflammation and Aging, Karolinska University Hospital, 17177, Stockholm, Sweden; g Institute of Public Health and Clinical Nutrition, University of Eastern Finland, 70211, Kuopio, Finland; h Ageing Epidemiology Research Unit, School of Public Health, Imperial College London, London, SW7 2AZ, United Kingdom; i Department of Neurobiology, Care Sciences and Society, Division of Family Medicine and Primary Care, Karolinska Institutet, 17177, Stockholm, Sweden; j Department of Clinical Neuroscience, Karolinska Institutet, 17177, Stockholm, Sweden; k Center for Cognitive and Computational Neurosceince (CCNP), Karolinska Institutet, 17177, Stockholm, Sweden

**Keywords:** Machine learning, Alzheimer`s disease, Primary health care, Predictive model, Diagnostic factors

## Abstract

**Background:**

Integrating machine learning with medical records offers potential for early detection of Alzheimer's disease (AD), enabling timely interventions.

**Objectives:**

This study aimed to evaluate the effectiveness of machine learning in constructing a predictive model for AD, designed to predict AD with data up to three years before diagnosis. Using clinical data, including prior diagnoses and medical treatments, we sought to enhance sensitivity and specificity in diagnostic procedures. A second aim was to identify the most important factors in the machine learning models, as these may be important predictors of AD.

**Design:**

The study employed Stochastic Gradient Boosting, a machine learning method, to identify diagnoses predictive of AD using primary healthcare data. The analyses were stratified by sex and age groups.

**Setting:**

The study included individuals within Region Stockholm, Sweden, using medical records from 2010 to 2022.

**Participants:**

The study analyzed clinical data for individuals over the age of 40. Patients with an AD diagnosis (ICD-10-SE codes F00 or G30) during 2010–2012 were excluded to ensure prospective modeling. In total, AD was identified in 3,407 patients aged 41–69 years and 25,796 patients aged over 69.

**Measurements:**

The machine learning model ranked predictive diagnoses, with performance assessed by the area under the receiver operating characteristic curve (AUC). Known and novel predictors were evaluated for their contribution to AD risk.

**Results:**

AUC values ranged from 0.748 (women aged 41–69) to 0.816 (women over 69), with men across age groups falling within this range.

Sensitivity and specificity ranged from 0.73 to 0.79 and 0.66 to 0.79, respectively, across age and gender groups. Negative predictive values were consistently high (≥0.954), while positive predictive values were lower (0.199–0.351).

Additionally, we confirmed known risk factors as predictors and identified novel predictors that warrant further investigation. Key predictors included medical observations, cognitive symptoms, antidepressant treatment, visit frequency, and vitamin B12/folic acid treatment.

**Conclusions:**

Machine learning applied to clinical data shows promise in predicting AD, with robust model performance across age and sex groups. The findings confirmed known risk factors, such as depression and vitamin B12 deficiency, while also identifying novel predictors that may guide future research. Clinically, this approach could enhance early detection and risk stratification, facilitating timely interventions and improving patient outcomes.

## 1 Introduction

Alzheimer's disease (AD) is a debilitating neurodegenerative disorder that impacts millions of individuals across the globe. The prevalence of AD is increasing with the growing population aged 65 and over, who are at higher risk [1]. Many individuals with dementia remain undiagnosed [2,3] or are diagnosed at an advanced stage of the disease [4]. A systematic review revealed a global undetected dementia rate of 61.7 % with factors such as being under 70, male, and diagnosed by general practitioners [5], underlining the potential utility of machine learning tools in primary care settings, where AD may not be considered despite present predisposing factors. Enhanced early diagnosis efforts are essential to address these disparities and improve dementia detection in community settings [6].

AD is a progressive disease with a prodromal stage in which the etiology is not completely understood. From a neuropathological perspective, AD is characterized by amyloidosis, tau pathology, and neurodegeneration. Neuropathological features of AD and subtle cognitive dysfunction have been associated with the presence of neurofibrillary tangles and senile plaques also in individuals not yet diagnosed with AD, suggesting that these processes may represent a prolonged preclinical stage of the disease [[7], [8], [9]]. Certain diagnoses and symptoms treated in primary care may also be predictors for AD [10,11].

Early detection of risk for AD may improve the possibility for deterring disease progression [12,13]. The prodromal stage of AD, where cognitive decline is detectable but not yet severe enough to warrant a diagnosis of dementia, typically represents the transition period between normal aging and full-blown AD. Identifying patients in this stage allows for timely treatments, and early administration of medications and lifestyle interventions has shown promise in slowing AD progression [13]. Modifiable risk factors such as stress, depression, diet, physical activity, and sleep patterns, can significantly impact cognitive health [14]. Recent advancements in drug development, such as targeted molecular therapies and disease-modifying treatments [15], indicate a shift towards interventions [14] capable of altering the progression of dementia [16].The reason for low diagnostic activity in the primary care is probably multifaceted with socioeconomic differences but may change as medications become more beneficial. Although there are treatments available for AD, their benefits are still limited and most effective when administered early. This may lead providers to question the value of diagnosis and management. Moreover, primary care physicians are often under pressure to manage a high volume of patients, which may limit the time available for in-depth screenings before AD becomes evident.

There has been a surge in efforts to identify individuals at risk for dementia using a variety of methods such as diagnostic blood tests, clinical markers and MRI-scans of the brain, to enable early intervention strategies [[17], [18], [19]]. Recent progress in artificial intelligence (AI) and machine learning methods has demonstrated significant potential in improving the accuracy and efficiency of clinical prediction models [20]. Among these techniques, gradient boosting has gained significant attention due to its capability to manage complex, high-dimensional data and uncover intricate relationships within large datasets [21,22]. This article focuses on the utilization of Stochastic Gradient Boosting (SGB) and its potential to pinpoint relevant diagnostic markers for AD detection within a primary care setting. Machine learning has previous been explored in relation to finding early signs predictive of AD using structural MRI scans, PET-scans of the brain [19,23,24] and EEG [25]. However, such methods may also be applied to the patterns in the clinical history of a patient. Machine learning models have the potential to identify high-risk individuals for dementia at a stage earlier than current assessment for dementia. Detection models that use easily accessed, clinical data may potentially lead to earlier and more cost-effective diagnoses in primary care settings.

The aim of this study is to assess the effectiveness of machine learning in constructing a predictive model for AD, designed to predict AD with data up to three years before diagnosis. Using clinical data, including prior diagnoses and medical treatments, we sought to enhance sensitivity and specificity in diagnostic procedures. A second aim is to identify the most important factors in the machine learning models, as these may be important predictors of AD.

## 2 Methods

### 2.1 Study design

This study was not registered in advance. We employed a case-control design, matched for sex and age, to construct a predictive model for AD utilizing a machine learning algorithm. The study used prospectively collected medical record data from the VAL-databases. The databases contain nearly all medical diagnoses, prescriptions and consultations in all care forms in Region Stockholm. Region Stockholm accounts for over one-fifth of Sweden's population, with more than 2.4 million residents, covering not only Stockholm but also its surrounding urban and rural areas. All diagnoses are coded according to the International Classification of Diseases, 10th Revision (ICD-10). To reflect available clinical data for doctors working in primary care, we collected data about diagnoses and visits registered at primary health care centers and about collected prescribed medications in the population of Region Stockholm.

### 2.2 Participants

The study population consisted of individuals aged 40 and above, registered at primary health care centers in Region Stockholm between 2010 and 2022. Individuals diagnosed with AD (ICD-10-SE codes F00 or G30) during 2010–2012 were excluded from the study.

Cases were defined as individuals diagnosed with AD (ICD-10-SE codes F00 or G30) between 2013 and 2022. These cases were categorized into two age groups: 41–69 years and over 69 years, and further classified by sex (male and female). Each case was matched with up to ten controls based on age and sex.

For each case, a control without an AD diagnosis was selected, ensuring that they had similar inclusion dates in the database and comparable observation periods.

### 2.3 Variables

We collected demographic information, including age and sex, as well as medical diagnoses classified by ICD codes, recorded within the three years prior to the index date. Additionally, we gathered data on the frequency of consultations at primary health care centers during two time periods: 6–18 months and 18–30 months before the index date. All diagnoses within the three-year period preceding the index date were compiled for each individual, focusing on the 2000 most commonly registered diagnoses in primary care. The index date was defined as six months prior to the AD diagnosis.

ICD codes for chronic diseases and conditions with similar clinical characteristics were grouped into common clinical categories, see Supplementary Table 1. A comparable method was applied for medications to ATC codes, where most codes consisted of one letter followed by two digits. Medications of particular interest were assigned codes with higher resolution, see Supplementary Table 2.

Some diagnostic codes can be confusing, for example “medical observations for suspected diseases and conditions”. This applies when a person has signs or symptoms requiring further investigation. Moreover, “other symptoms and signs involving cognitive functions and awareness” is used for unexplained symptoms and abnormal clinical and laboratory findings, “encounter for general examination without complaint, suspected or reported diagnosis” covers routine check-ups and suspected conditions. “Persons encountering health services for other counseling and medical advice. Not elsewhere classified” applies to visits for a specific advice, limited care, or service for a current condition.

Outpatient and inpatient visits were combined, with multiple visits on the same date counted as a single visit. We chose not to include the frequency of visits during the last six months prior to the index date, due to the typically intensive evaluations required to confirm a dementia diagnosis during this period. Prescription drug dispensations were recorded based on Anatomical Therapeutic Chemical (ATC) codes, where at least two dispensations within a 12-month period before the index date were considered indicative of consumption and included as variables.

It is crucial to avoid providing the machine learning model with information that could directly aid in predicting the outcome, as this is already factored into the physician's diagnostic process. Therefore, certain variables were excluded from the analyses, including vascular dementia (F01), dementia in other diseases classified elsewhere (F02), unspecified dementia (F03), mild cognitive impairment and brain damage (F06), and medications for dementia (N06D).

### 2.4 Statistical analyses

Categorical data are presented as frequencies and percentages, denoted as n ( %). We applied SGB, a machine learning technique that integrates boosting and randomization to build robust predictive models [21], using R version 4.2.2. The SGB model was employed to identify key diagnoses indicative of dementia cases in primary healthcare settings. SGB is known for its high predictive accuracy and the ability to provide insight into important factors, along with odds ratios of marginal effects [[26], [27], [28]].

The model parameters included up to 20,000 trees, 5 interactions, a shrinkage (learning rate) of 0.001, a minimum of 10 observations per terminal node, and a subsampling rate (bag fraction) of 0.5. The optimal number of trees was determined through 10-fold cross-validation. Analyses were stratified by sex due to known differences in risk factors and diagnostic patterns between men and women and portrayed by area under the receiver operating curves (AUC).

The dataset was divided into men and women, as well as into two age groups: younger adults (41–69 years) and older adults (over 69 years). Each group was further split into 70 % training and 30 % test sets, ensuring blinding but a balanced representation of AD cases across both sets. Variables occurring at least 25 times were retained for analysis: 176 variables for men aged 41–69, 486 for men over 70, 234 for women aged 41–69, and 563 for women over 70.

The SGB model ranked the most influential diagnoses associated with newly diagnosed AD. Odds ratios were calculated from the predicted probabilities of AD, derived from the partial dependence plots of the gradient boosting model. ORME refers to the odds ratios for the marginal effects.

As a measure of feature importance, we used normalized relative influence (NRI).

This study follows the TRIPOD+AI reporting guidelines to ensure transparency and reproducibility in the development and validation of the machine learning model [29]. A completed TRIPOD+AI checklist is provided, Supplementary Table 3.

### 2.5 Ethics

All data was pseudonymized. The study was approved by the Ethics Review Board in Stockholm (2021–01016 with later amendments 2023–07166–02 and 2024–05462–02). The data included in this study can be available for research purposes after ethical approval from Stockholm Region at halsodata.rst@regionstockholm.se; or through collaborations with us.

## 3 Results

In this study, a total of 3407 patients (1961 women and 1446 men) aged 41–69 years, and 25,796 patients (16,372 women and 9424 men) 70 years and older were identified as having AD. Baseline characteristics among cases and controls are shown in Table 1.

**Table 1:** The baseline characteristics in patients and controls.

	**Alzheimers=No**		**Alzheimers=Yes**	
	**Female (N=174,635)**	**Male (N=117,445)**	**Female (N=18,336)**	**Male (N=10,872)**
**Age**				
Mean (SD)	77.7 (7.98)	77.8 (7.71)	81.6 (7.92)	80.1 (7.81)
Median [Min, Max]	76.0 [41.0, 115]	77.0 [41.0, 119]	83.0 [41.0, 119]	81.0 [41.0, 106]
**Diabetes**				
No	166,739 (95.5 %)	110,414 (94.0 %)	17,030 (92.9 %)	9623 (88.5 %)
Yes	7896 (4.5 %)	7031 (6.0 %)	1306 (7.1 %)	1249 (11.5 %)
**Hypertension**				
No	142,264 (81.5 %)	98,556 (83.9 %)	13,043 (71.1 %)	7754 (71.3 %)
Yes	32,371 (18.5 %)	18,889 (16.1 %)	5293 (28.9 %)	3118 (28.7 %)
**Coronary Heart Disease**				
No	171,339 (98.1 %)	113,381 (96.5 %)	17,773 (96.9 %)	10,218 (94.0 %)
Yes	3296 (1.9 %)	4064 (3.5 %)	563 (3.1 %)	654 (6.0 %)
**Stroke Cerebrovascular Disease**				
No	172,420 (98.7 %)	115,414 (98.3 %)	17,952 (97.9 %)	10,428 (95.9 %)
Yes	2215 (1.3 %)	2031 (1.7 %)	384 (2.1 %)	444 (4.1 %)
**COPD**				
No	170,474 (97.6 %)	114,874 (97.8 %)	17,820 (97.2 %)	10,512 (96.7 %)
Yes	4161 (2.4 %)	2571 (2.2 %)	516 (2.8 %)	360 (3.3 %)
**Depression**				
No	171,404 (98.1 %)	116,303 (99.0 %)	17,168 (93.6 %)	10,441 (96.0 %)
Yes	3231 (1.9 %)	1142 (1.0 %)	1168 (6.4 %)	431 (4.0 %)
**Anxiety Disorders**				
No	171,507 (98.2 %)	116,509 (99.2 %)	17,376 (94.8 %)	10,599 (97.5 %)
Yes	3128 (1.8 %)	936 (0.8 %)	960 (5.2 %)	273 (2.5 %)
**Alcohol Dependence**				
No	174,512 (99.9 %)	117,251 (99.8 %)	18,282 (99.7 %)	10,803 (99.4 %)
Yes	123 (0.1 %)	194 (0.2 %)	54 (0.3 %)	69 (0.6 %)
**Number of Visits (6–18 Months)**				
Mean (SD)	12.1 (30.7)	10.1 (26.7)	43.3 (68.2)	37.4 (60.2)
Median [Min, Max]	3.00 [0, 363]	1.00 [0, 362]	20.0 [0, 361]	19.0 [0, 362]
**Number of Visits (18–30 Months)**				
Mean (SD)	11.3 (29.5)	9.63 (25.7)	24.3 (50.0)	21.9 (44.6)
Median [Min, Max]	2.00 [0, 363]	1.00 [0, 364]	10.0 [0, 362]	10.0 [0, 361]
**Dementia (F02)**				
No	174,608 (100.0 %)	117,408 (100.0 %)	18,322 (99.9 %)	10,858 (99.9 %)
Yes	27 (0.0 %)	37 (0.0 %)	14 (0.1 %)	14 (0.1 %)
**Dementia (F03)**				
No	174,146 (99.7 %)	117,171 (99.8 %)	17,829 (97.2 %)	10,519 (96.8 %)
Yes	489 (0.3 %)	274 (0.2 %)	507 (2.8 %)	353 (3.2 %)
**Dementia (F06)**				
No	174,053 (99.7 %)	117,034 (99.7 %)	17,319 (94.5 %)	10,154 (93.4 %)
Yes	582 (0.3 %)	411 (0.4 %)	1017 (5.5 %)	718 (6.6 %)
**B12 & Folic Acid**				
No	155,153 (88.8 %)	106,011 (90.3 %)	14,143 (77.1 %)	8449 (77.7 %)
Yes	19,482 (11.2 %)	11,434 (9.7 %)	4193 (22.9 %)	2423 (22.3 %)
**Vitamines**				
No	169,279 (96.9 %)	114,678 (97.6 %)	17,276 (94.2 %)	10,288 (94.6 %)
Yes	5356 (3.1 %)	2767 (2.4 %)	1060 (5.8 %)	584 (5.4 %)

The models demonstrated strong performance on the test datasets, with an AUC of 0.816 (CI 0.795–0.837) for women aged 41–69 years and 0.748 (CI 0.740–0.757) for women aged 70 years and older. Similarly, for men, the AUC was 0.775 (CI 0.747–0.804) for those aged 41–69 years and 0.753 (CI 0.741–0.764) for those aged 70 years and older.

**Fig. 1:**
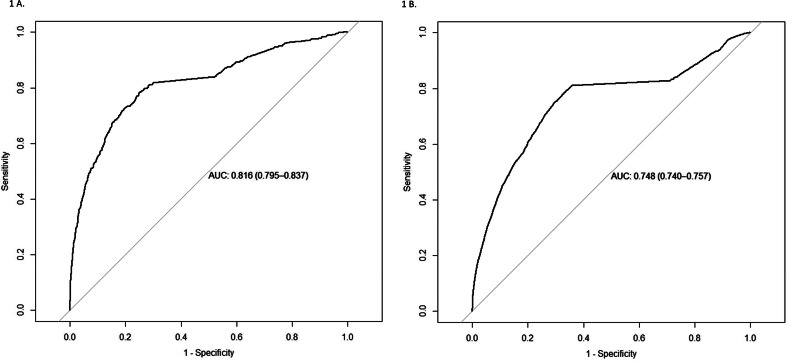
A and B Receiver operating characteristic (ROC) curves for the test dataset of women.

**Fig. 2:**
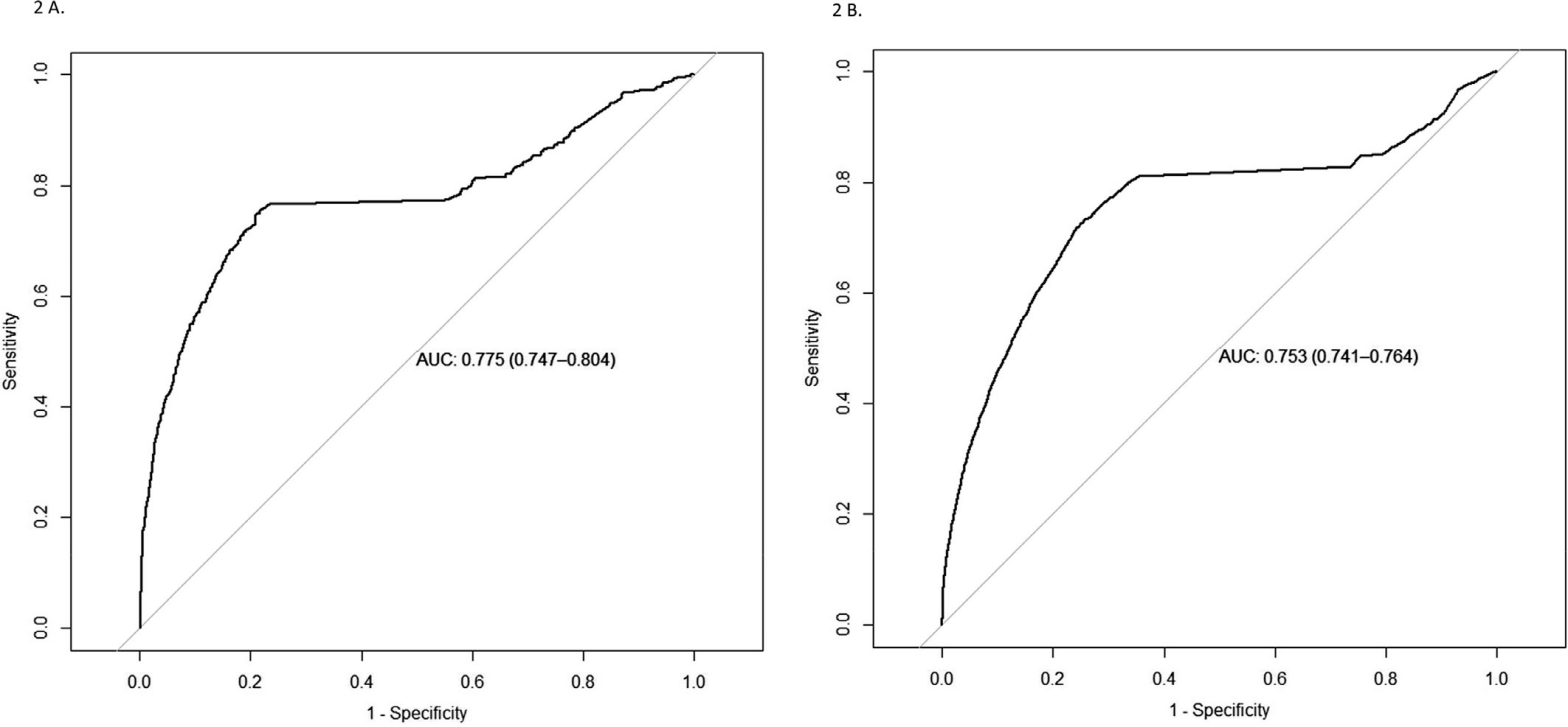
A and B Receiver operating characteristic (ROC) curves for the test dataset of men.

The predictive performance of the SGB model is presented in Table 2 and illustrated in Fig. 1, Fig. 2. Using 12,000 to 20,000 decision trees, the model's sensitivity and specificity showed slight variations across demographic groups. Among women aged 41–69 years, the model achieved a sensitivity of 0.78 and a specificity of 0.75, while for women over 69 years, sensitivity was 0.79 and specificity was 0.66. For men, those aged 41–69 years had a sensitivity of 0.75 and a specificity of 0.79, whereas men over 69 years demonstrated a sensitivity of 0.73 and a specificity of 0.74.

**Table 2:** Confusion matrix for predicting Alzheimer's Disease in women and men, aged 41–69 years old and >70 years old, using optimal stochastic gradient. Predictions based on test data.

	Observed		
Predicted	No Alzheimer`s	Alzheimer`s	Total
Age 41–69			
Women without Alzheimer`s disease	4023	124	4147
Women with Alzheimer`s disease	1332	447	1779
Women total	5355	571	5926
Positive predictive value		0.251	
Negative predictive value	0.970		
Men without Alzheimer`s disease	2389	115	2504
Men with Alzheimer`s disease	637	344	981
Men total	3026	459	3485
Positive predictive value		0.351	
Negative predictive value	0.954		
			
Age >70			
Women without Alzheimer`s disease	31,006	1031	32,037
Women with Alzheimer`s disease	15,958	3969	19,927
Women total	46,964	5000	51,964
Positive predictive value		0.199	
Negative predictive value	0.968		
Men without Alzheimer`s disease	23,969	760	24,729
Men with Alzheimer`s disease	8219	2061	10,280
Men total	32,188	2821	35,009
Positive predictive value		0.200	
Negative predictive value	0.969

Receiver operating characteristic (ROC) curves for the test dataset of women, generated using the stochastic gradient boosting models. Panel A shows results for women aged 41–69 years, and Panel B for women >70 years.

Receiver operating characteristic (ROC) curves for the test dataset of men, generated using the stochastic gradient boosting models. Panel A shows results for men aged 41–69 years, and Panel B for men >70 years.

For men aged 41–69 years, the model achieved a positive predictive value (PPV) of 0.351 and a negative predictive value (NPV) of 0.954. In men aged 70 and older, the PPV was 0.200, while the NPV was 0.969. Among women aged 41–69 years, the model showed a PPV of 0.251 and an NPV of 0.970. For women aged 70 and older, the PPV was 0.199, with an NPV of 0.968. These results suggest that the model performed more effectively in ruling out AD than in predicting its presence.

The variables predicting the onset of AD in both women and men are presented in Table 3, with only variables having an NRI greater than 1 included in the Table. For both genders, key factors influencing AD diagnosis included medical observations for suspected diseases and conditions that were ruled out (ORME: 5.8 in women aged 41–69 years, 3.5 in women over 70, 7 in men aged 41–69, and 4.3 in men over 70), and cognitive symptoms such as other signs involving cognitive functions and awareness (ORME: 60.6 in women aged 41–69, 25.5 in women over 70, 98.6 in men aged 41–69, and 29.5 in men over 70).

Antidepressant treatment also emerged as a significant factor (ORME: 3.8 in women aged 41–69, 3.1 in women over 70, 7 in men aged 41–69, and 3.4 in men over 70). Additionally, the number of healthcare visits 6–18 months prior to diagnosis (ORME: 15.1 in women aged 41–69, 24.9 in women over 70, 7.9 in men aged 41–69, and 9.7 in men over 70) and vitamin B12 or folic acid treatment (ORME: 2.7 in women aged 41–69, 3.1 in women over 70, 5 in men aged 41–69, and 3.5 in men over 70) were influential predictors (Table 3a, b).

**Table 3a:** Variables predicting the onset of Alzheimer's disease in women 41–69 years old and >70 years old, were identified. Only variables with a normalized relative influence (NRI) greater than 1 were included, along with the odds ratios for the marginal effects (ORME) of Alzheimer's disease.

Variable	ICD-10 code/ATC	NRI ( %)	OR_ME_
41–69 years			
Medical observation for suspected diseases and conditions ruled out	Z03	19,72	5,8
Other symptoms and signs involving cognitive functions and awareness	R41	14,09	60,6
Antidepressants	N06A	10,73	3,8
Number of visits 6–18 months before diagnosis		5,92	15,1
Vitamin B12 and folic acid	B03B	5,27	2,7
Vitamines (combinations)	A11	2,33	2,3
Other anxiety disorders	F41	1,82	1,7
Encounter for general examination without complaint, suspected or reported diagnosis	Z00	1,66	2,1
Depressive episode	F32	1,52	1,7
Reaction to severe stress, and adjustment disorders	F43	1,28	1,9
		1,28	0,8
Unspecified urinary incontinence	R32	1,20	5,2
Sedatives, tranquillizers	N05CF	1,16	0,9
Diuretics (aldosterone agonists, thiazide diuretics, and loop diuretics)	C03	1,13	0,8
Hypothyroidism (subclinical hypothyroid, hypothyroidism, other thyroid disease)	E02, E03, E07	1,08	1,6
Alcohol related disorders	F10	1,00	3,1
			
> 70 years			
Other symptoms and signs involving cognitive functions and awareness	R41	19,19	25,5
Number of visits 6–18 months before diagnosis		12,62	24,9
Medical observation for suspected diseases and conditions ruled out	Z03	11,58	3,5
Vitamin B12 and folic acid	B03B	11,23	3,1
Antidepressants	N06A	11,19	3,1
Anticoagulants	B01AA+B01AB+B01AC+B01AD+B01AX	1,85	1,4
Drugs for peptic ulcer and gastro-esophageal reflux disease	A02B	1,73	0,9
Cough and cold preparations	R05	1,33	0,9
Number of visits 18–30 months before diagnosis		1.32	0.6
Sedatives. tranquillizers	N05BA+N05BC+N05BD+N05BC+N05BE+N05BX	1.23	1
Other symptoms and signs involving general sensations and perceptions	R44	1.07	5.7
Opioids	N02A	1.04	1

**Table 3b:** Variables predicting the onset of Alzheimer's disease in men 41–69 years old, and >70 years old, were identified. Only variables with a normalized relative influence (NRI) greater than 1 were included, along with the odds ratios for the marginal effects (ORME) of Alzheimer's disease.

Variables	ICD-10 code/ ACT	NRI ( %)	OR_ME_
41–69 years			
Medical observation for suspected diseases and conditions ruled out	Z03	18.90	7
Antidepressants	N06A	12.82	6
Other symptoms and signs involving cognitive functions and awareness	R41	12.03	98.6
Vitamin B12 and folic acid	B03B	10.20	5
Number of visits 6–18 months before diagnosis		6.09	7.9
Vitamines	A12	2.42	3
Antibiotics	J01	1.61	0.9
Persons encountering health services for other counseling and medical advice. not elsewhere classified	Z71	1.60	2.6
Number of visits 18–30 months before diagnosis		1.30	0.8
Drugs for peptic ulcer and gastro-esophageal reflux disease	A02B	1.16	0.9
Parkinson's disease	N04	1.08	2.2
Anticoagulants	B01AA+B01AB+B01AC+B01AD´B01AX	1.03	1.2
Stroke & transient ischaemic attack	I60- I69, I74	1.03	2.4
			
> 70 years			
Medical observation for suspected diseases and conditions ruled out	Z03	15.84	4.3
Other symptoms and signs involving cognitive functions and awareness	R41	15.72	29.5
Vitamin B12 and folic acid	B03B	14.09	3.5
Number of visits 6–18 months before diagnosis		7.67	9.7
Antidepressants	N06A	6.95	3.4
Anticoagulants	B01AA+B01AB+B01AC+B01AD´B01AX	2.56	1.6
Drugs for peptic ulcer and gastro-esophageal reflux disease	A02B	1.23	0.9
Antibiotics	J01	1.16	1
Vitamines (combinations)	A11	1.05	1.6

## 4 Discussion

Our machine learning models detected AD with good accuracy; AUC values above 80 % are generally considered clinically relevant [30]. In our study, AUC values ranged from 0.816 in women aged 41–69 years to 0.748 in women aged 70 years, with men in both age groups falling within this range.

Additionally, we confirmed known risk factors as predictors and identified novel predictors that warrant further investigation. The following variables were consistently identified and validated across all groups as predictive of AD: medical observation for suspected diseases and conditions ruled out, other symptoms and signs related to cognitive functions and awareness, antidepressant treatment, number of healthcare visits 6–18 months before diagnosis, and prescribed vitamin B12 and folic acid.

While the AUCs are promising, the PPVs are consistently low across all models. This suggests that while the models are effective in ruling out AD, they are less reliable for confirming its presence. However, identifying at least one in four or five individuals as having AD may still be useful for predicting high risk. Since NPVs exceed 95 % in all models, they can effectively rule out AD, ensuring that few cases are missed. Furthermore, a high PPV is less critical, as emerging laboratory tests for amyloids and tau will enable more precise confirmation of diagnoses in those identified by the machine learning model

The false discovery rate (FDR) of the current instrument ranged from approximately 20.6 % to 26.9 % across different subgroups, meaning that 20–27 % of the positive predictions made by the model were false positives. In practical terms, if the model were implemented in the Swedish primary care system, this rate would imply that a significant proportion of patients flagged as high risk may not actually have the condition, potentially resulting in unnecessary follow-up testing or clinical interventions. Considering Sweden's population and the potential widespread use of the instrument, even a modest FDR could lead to a substantial number of false positives in absolute terms. Therefore, further refinement of the model, particularly improving its PPV and specificity, is essential before broad clinical implementation. Additionally, strategies to minimize the clinical burden of false positives should be explored, including targeted follow-up protocols and streamlined confirmatory diagnostic tests.

Globally, AD detection rates are notably lower in individuals under 70 years, a group for which our models performed particularly well. Previous research has validated the effectiveness of machine learning in AD prediction, highlighting the importance of early diagnosis in mitigating the disease's impact. For instance, studies using the Open Access Series of Imaging Studies (OASIS) dataset have reported accuracy rates between 83 % and 96 % with various machine learning techniques, including Decision Trees, Random Forests, and Gradient Boosting [31,32].^.^ However, these models, which primarily rely on neuroimaging data, face limitations in real-world applicability and are challenging to implement in routine screening programs.

To address these limitations, multimodal approaches integrating clinical data, MRI segmentation, and psychological assessments have been developed, significantly improving both prediction accuracy and model interpretability. For example, a study using nine machine learning models reported that the Random Forest classifier achieved the highest 10-fold cross-validation accuracy of 98.81 % [33]. Additionally, a systematic review of 23 articles underscores the importance of explainable artificial intelligence in improving the reliability and trustworthiness of AI-based AD predictions, which is essential for improving clinical decision support systems for AD prognosis [34]. The contribution of the present study is that it demonstrates how variables from electronic patient records can be effectively used to detect AD, offering a potentially valuable tool for early diagnosis.

Our findings identified several factors commonly associated with AD that may serve as valuable predictors. For instance, investigations aimed at ruling out serious conditions could be complemented with biomarker analyses to detect AD in time. Additionally, newly diagnosed depression may also be a relevant warning sign of AD. Previous research, including our own, has demonstrated that both depression and chronic stress are risk factors for dementia [[35], [36], [37]], and our findings replicated the association between depression treatment and AD.

This study also found an association between the prescription and collection of vitamins and an increased risk of AD. The underlying rationale for the association between vitamins and AD could be multifaceted. Patients prescribed vitamins - particularly those linked to cognitive health, such as B12 and folic acid, may already displaying symptoms of cognitive decline or related health concerns, prompting their prescription [38]. Moreover, these vitamins are often used to correct deficiencies that could exacerbate cognitive impairment and accelerate AD progression [39,40]. However, this is a limitation in this study, certain diagnoses, such as vitamin B12 deficiency, are considered when clinicians suspect dementia due to the established link between vitamin B12 and AD [41]. Since excluding vitamin B12 deficiency is a routine step in AD diagnostics, and high-dose vitamin B12 treatment can mitigate neurological symptoms, including cognitive impairment, this may impact the recorded frequency of such diagnoses in medical records.

A key strength of this study is the use of a comprehensive database that includes the entire resident population of Region Stockholm, more than 2 million individuals. The extensive dataset allows for a broad and inclusive analysis of AD prediction across diverse demographic groups. Machine learning algorithms provide a powerful exploratory approach, uncovering novel predictive associations that traditional models might overlook. Unlike human cognition, these algorithms are unbiased by socio-economic status, identifying predictors objectively and signaling clinicians for further investigation. Machine learning assistance in identifying AD allows for the timely initiation of potential disease-modifying treatments, which will be particularly crucial as new therapies become available in the coming years.

This study has several limitations. Traditional prognostic models typically rely on established risk factors and their theoretical relationships with outcomes. In contrast, AI models like ours uncover novel predictive associations that require external validation. The predictors identified in our model need further confirmation before they can be confidently implemented in clinical settings. Another limitation is the potential for reverse causation. For example, the frequency of healthcare visits, medication usage, and certain diagnoses might be influenced by an individual's likelihood of being diagnosed with AD, rather than directly contributing to the onset of AD. This complicates the interpretation of some predictive factors.

Another potential limitation of the study is the model's applicability to other countries or healthcare systems. Its relevance may be limited to Sweden, and further investigation are therefore needed to replicate the model in different settings.

While our model shows promise, its practical impact on guiding clinical decisions and informing further investigations has yet to be demonstrated. Some risk factors may be indicative of doctors already investigating dementia. We excluded variables that were clearly associated with dementia and AD, such as other types of dementia, brain damage, and mild cognitive impairment, as these would not be helpful in predicting AD when it has already been suspected or diagnosed. Future research should aim to validate these findings, assess the model's clinical applicability, and explore the use of other clinical variables in predicting AD through machine learning. Incorporating laboratory results and additional risk factors, such as lifestyle factors, from electronic health records could probably enhance accuracy. Future research should enhance variable selection and apply advanced machine learning techniques to bridge the gap between prediction models and clinical use.

## 5 Conclusion

In this unbiased study design, we verified known risk factors as predictors, such as depression and vitamin B12, and discovered novel predictors that warrant further investigations, such as medical observation for suspected diseases and conditions, other symptoms and signs involving cognitive functions and awareness and number of visits to doctors in primary care 6–18 months before diagnosis.

The consistent identification of key predictors across various models underscores the robustness of our findings. The present study suggests that clinical historical data may contribute to increased sensitivity and specificity in identification of individuals that will develop dementia, especially AD. Our results demonstrate that machine learning models based on data in the electronic patient records may be effective in predicting AD, especially among individuals who may not clearly describe their symptoms and in younger populations where diagnosis can be challenging.

## Funding

Axel C Carlsson received funding for his salary from Region Stockholm (FoUI-973,001) and for the whole project from the Swedish research council (2023–05810).

## Consent statement

Consent was not applicable/necessary.

## Availability of data and materials

The data from this study can be accessed for research by qualified researchers who have been trained in confidentiality protocols for human subjects, following ethical approval from Region Stockholm at halsodata.rst@regionstockholm.se. Analytical code and programs can be obtained from axel.carlsson@ki.se.

## Declaration of Generative AI and AI-assisted technologies in the writing process

During the preparation of this work the authors used ChatGPT 3.5 for linguistic accuracy. After using this tool/service, the authors reviewed and edited the content as needed and take full responsibility for the content of the publication.

## CRediT authorship contribution statement

**Johanna Wallensten:** Writing – original draft, Investigation. **Caroline Wachtler:** Writing – review & editing, Conceptualization. **Nenad Bogdanovic:** Writing – review & editing, Investigation. **Anna Olofsson:** . **Miia Kivipelto:** Writing – review & editing, Investigation. **Linus Jönsson:** Writing – review & editing, Methodology. **Predrag Petrovic:** Writing – review & editing, Investigation. **Axel C. Carlsson:** Writing – review & editing, Supervision, Methodology, Conceptualization.

## Declaration of competing interest

The authors declare the following financial interests/personal relationships which may be considered as potential competing interests: Axel C Carlsson reports financial support was provided by Region Stockholm. Axel C Carlsson reports financial support was provided by Swedish Research Council. If there are other authors, they declare that they have no known competing financial interests or personal relationships that could have appeared to influence the work reported in this paper.

## Appendix Supplementary materials

Image, application 1
