# Supplementary material for: Machine learning to detect Alzheimer's disease with data on drugs and diagnoses
Source: J Prev Alzheimers Dis. 2025 Mar 8;12(5):100115. doi: 10.1016/j.tjpad.2025.100115 (PMC12184014; doi:10.1016/j.tjpad.2025.100115)
Supplement: Supplementary file 1 [file mmc1.docx]

**Supplementary Table 1**

| **Disease or condition** | **ICD-10 codes** | **ICD-10 that were merged** |
| --- | --- | --- |
| **Arterial conditions** | I77  I79 | Other disorders of arteries and arterioles  Disorders of arteries, arterioles and capillaries in diseases classified elsewhere |
| **Arthritis** | M50  M51  M52 | Cervical disc disorders  Thoracic, thoracolumbar, and lumbosacral intervertebral disc disorders  Other dorsopathies |
| **Chronic liver disease** | K70  K72  K74  K75  K76K77 | Alcohol-related liver disease  Hepatic failure, not elsewhere classified  Fibrosis and cirrhosis of liver  Other inflammatory liver diseases  Other diseases of liver  Liver disorders in diseases classified elsewhere |
| **Chronic obstructive pulmonary disease** | J41  J42  J43  J44 | Simple and mucopurulent chronic bronchitis  Unspecified chronic bronchitis  Emphysema  Other chronic obstructive pulmonary disease |
| **Chronic renal disease** | I12  N18  N19  N05  N25 | Hypertensive renal disease w/ renal failure  Chronic renal disease  Unspecified chronic renal disease  Unspecified nephritic syndromes  Disorders resulting from impaired renal tubular function |
| **Coronary heart disease** | I20  I21  I22  I23  I24  I25 | Angina pectoris  Acute myocardial infarction  Subsequent myocardial infarction  Complications to myocardial infarction  Acute ischemic heart disease  Chronic ischemic heart disease |
| **COVID-19** | U07  U08  U09  U11  U12 | COVID-19, virus identified in laboratory (U07.1)  COVID-19 (U07.2)  Personal history of COVID-19, unspecified (U08.9)  Post-COVID condition, unspecified (U09.9)  Need for immunization against COVID-19, unspecified (U11.9)  COVID-19 vaccines causing adverse effects in therapeutic use, unspecified (U12.9) |
| **Diabetes** | E10  E11  E12  E13  E14 | Type 1 Diabetes mellitus  Type 2 Diabetes mellitus  Malnutrition-related diabetes mellitus  Other specified diabetes mellitus  Unspecified diabetes mellitus |
| **Dyspepsia (current)** | K29  K30 | Chronic superficial gastritis (K 29.3)  Chronic unspecified gastritis (K29.4)  Chronic atrophic gastritis (K29.5)  Other specified gastritis (K29.6)  Unspecified gastritis (K29.7)  Functional dyspepsia |
| **Encephalitis** | G04  G05  B86  B94 | Encephalitis, myelitis and encephalomyelitis  Encephalitis, myelitis and encephalomyelitis in diseases classified elsewhere  Scabies  Sequelae of viral encephalitis |
| **Glaucoma** | H40  H42 | Glaucoma  Glaucoma in diseases classified elsewhere |
| **Hearing loss** | H91  H92 | Other and unspecified hearing loss  Otalgia and effusion of ear |
| **Hypertension** | I10  I11  I12  I13  I15 | Essential hypertension,  Hypertensive heart disease  Hypertensive kidney disease,  Hypertensive heart and kidney disease  Secondary hypertension |
| **Inflammatory bowel disease** | K50  K51 | Crohn disease  Ulcerative colitis |
| **Myocarditis** | I40  I41 | Acute myocarditis  Myocarditis in diseases classified elsewhere |
| **Painful conditions** | K58  R52  B02  G09  G35  G50  G53  G54  G55  G56  G57  G58  G59  G60  G61  G62  G63  G95 | Irritable bowel syndrome with diarrhea (K580)  Irritable bowel without diarrhea (I589)  Chronic treatment resistant pain (R521)  Other chronic pain (R522)  Persistent pain, nocioceptive (R522A)  Persistant pain, neuropathic (R522B)  Persistent pain, of unclear etiology  Herpes zoster  Sequelae of inflammatory diseases of central nervous  Multiple sclerosis  Trigeminal nerve disease  Cranial nerve disorders in diseases classified elsewhere  Nerve root and plexus disorders  Nerve root and plexus compressions in diseases classified elsewhere  Mononeuropathies of upper limb  Mononeuropathies of lower limb  Other mononeuropathies  Mononeuropathy in diseases classified elsewhere  Hereditary and idiopathic neuropathy  Inflammatory polyneuropathy  Other and unspecified polyneuropathies  Polyneuropathy in diseases classified elsewhere  Other and unspecified diseases of spinal cord |
| **Prostate disorders** | N40  N41  N42 | Prostate enlargement  Prostatitis  Other prostate disease |
| **Various dermatitis including psoriasis** | L20  L23  L24  L25  L26  L30  L40 | Atopic dermatitis  Allergic contact dermatitis  Irritative contact dermatitis  Unspecified contact dermatitis  Exfoliative dermatitis  Other and unspecified dermatitis  Psoriasis |
| **Rheumatoid arthritis, other inflammatory polyarthropathies, and systematic connective tissue disorders** | M05  M06  M07  M08  M09  M10  M11  M12  M30  M31  M32  M33  M34  M35 | Seropositive rheumatoid arthritis  Other rheumatoid arthritis  Psoriatic and entheropahtic arthropathies  Juvenile rheumatoid arthritis  Juvenile arthritis in diseases classified elsewhere  Gout  Other crystal arthropathies  Other and unspecified arthropathy  Polyarteritis nodosa and related conditions  Other necrotizing vasculopathies  Systemic lupus erythematosus (SLE)  Other dermatomysitis  Systemic sclerosis (scleroderma)  Other systemic involvement of connective tissue |
| **Specified z-codes of interest** | Z11  Z20  Z21  Z28  Z29  Z68  Z71  Z94  Z99 | Screening for infectious and parasitic diseases  Contact with and (suspected) exposure to communicable diseases  Asymptomatic human immunodeficiency virus [HIV] infection status  No vaccination due to contraindication (Z28.0)  Isolation at hospital ore care facility (Z29.0)  Body mass index (BMI) 30-39  Person with feared health complaint in whom no diagnosis is made without diagnosis (Z71.1)  Transplanted organ and tissue status  Dependence on enabling machines and devices, not elsewhere classified |
| **Stroke and transient ischaemic attack** | I60  I61  I62  I63  I64  I65    I66    I67  I68  I69  I74 | Nontraumatic subarachnoidhemorrhage  Nontraumatic intracerebral hemorrhage  Other and unspecified nontraumatic intracranial hemorrhage  Cerebral infarction  Stroke, not specified as hemorrhage or infarction  Occlusion and stenosis of precerebral arteries, not resulting in cerebral infarction  Occlusion and stenosis of cerebral arteries, not resulting in cerebral infarction  Other cerebrovascular disease  Cerebrovascular disorders in diseases classified elsewhere  Sequelae of cerebrovascular disease  Arterial embolism and thrombosis |
| **Thyroid disorders I** | E01  E04  E05  E06 | Iodine-deficicency related throid disorders and allied conditions  Other nontoxic goitre  Thyreotoxicosis (hyperthyroidism)  Thyroiditis |
| **Thyroid disorders II** | E02  E03  E07 | Subclinical iodine-deficiency hypothyroidism  Other hypothyroidism  Other thyroid disease |
| **Venous thromboembolism** | I26  I27  I80  I82 | Pulmonary embolism  Other pulmonary heart diseases  Phlebitis and thrombophlebitis  Other venous embolism and thrombosis |
| **Viral hepatitis** | B15  B16  B17  B18  B19 | Acute hepatitis A  Acute Hepatitis B  Other acute viral hepatitis  Chronic viral hepatitis  Unspecified viral hepatitis |
| **Upper respiratory tract infections** | J01  J02  J03  J06J20 | Acute sinusitis  Acute pharyngitis  Acute tonsillitis  Acute respiratory infection  Acute bronchitis |
| **Lower respiratory tract infection** | J15  J18  J22 | Bacterial pneumonia, not elsewhere classified  Pneumonia, organism unspecified  Acute lower respiratory tract infection |
| **Ear infections** | H65  H66  H68 | Non-suppurative otitis media  Suppurative and unspecified otitis media  Eustachian salpingitis and infection |

Supplementary Table 1: Diagnoses that were merged. All other ICD-10 codes were used as individual features with one letter and two digits.

**Supplementary Table 2**

| **Medication** | **ATC-codes of interest in higher resolution** |
| --- | --- |
| **A** |  |
| Antacids | A02A |
| Drugs for peptic ulcers and gastro-esophagal reflux disease (GERD) | A02B |
| Insulin | A10A |
| Metformin | A10BA |
| Sulfonylureas | A10BB |
| Dipeptidyl peptidase 4 (DPP-4) inhibitors | A10BH |
| Glucagon-like peptide-1 (GLP-1) analogues | A10BJ |
| Sodium-glucose cotransporter 2 (SGLT2) inhibitors | A10BK |
| **B** |  |
| Direct thrombin inhibitors  Direct factor Xa inhibitors | B01AE  B01AF |
| Iron preparations | B03A |
| Vitamin B12 and folic acid | B03B |
| **C** |  |
| Vasodilators used in cardicac diseases (only nitrates registered) | C01D |
| HMG CoA reductase inhibitors (statins)  Other lipid modifying agents  Combinations of various lipid modifying agents | C10AA  C10AX  C10BA |
| Hormonal contraceptives for systemic use | G03A |
| Androgens | G03B |
| Androgens and female sex hormones in combination | G03E |
| Progestogens and estrogens in combinations | G03F |
| Gonadotropins and other ovulations stimulants | G03G |
| Antiandrogens | G03H |
| Other sex hormones and modulators of the genital system | G03X |
| Drugs for urinary frequency and incontinence | G04BD |
| Drugs used in erectile dysfunction | G04BE |
| **H** |  |
| Corticosteroids for systemis use, plain | H02A |
| Thyroid preparations | H03A |
| **M** |  |
| Non-steroidal antiinflammatory drugs (NSAID) | M01A |
| **N** |  |
| Opioids | N02A |
| Salicylic acid and derivatives | N02BA |
| Paracetamol (only drug registered) | N02BE |
| Gabapentinoids | N02BF |
| Other analgesics and anipyretics | N02BG |
| Antipsychotics | N05A |
| Hydroxyzine (Atarax) | N05BB |
| Bensodiazepine-related drugs | N05CF |
| Melatoninreceptor-antagonists | N05CH01 |
| Other hypnotics and sedatives | N05CM06 |
| Antidepressants | N06A |
| Psychostimulants, agents used for ADHD and nootropics | N06B |
| Psycholeptics and psychoanaleptics in combination | N06C |
| Anti-dementia drugs | N06D |
| **R** |  |
| Adrenergic, inhalants  Other drugs for obstructive airways diseases, inhalants | R03A  R03B (merged) |
| Adrenergics for systemic use  Other systemic drigs for obstruct airway diseases | R03C  R03D (merged) |
| Phenothiazine derivatives | R06AD |

Supplementary Table 2: Medication (ATC-codes) of interest ATC-codes, either in higher resolution or merged

**Supplementary Table 3**

| **ITEM** | **#** | **Row in the manuscript the topic starts** |
| --- | --- | --- |
| Title | 1 | 1 |
| Abstract | 2 | 51 |
| Background | 3a | 120 |
|  | 3b | 130 |
|  | 3c | 127 |
| Objectives | 4 | 152 |
| Data | 5a | 172 |
|  | 5b | 174 |
| Participants | 6a | 172 |
|  | 6b | 177 |
|  | 6c | 195 |
| Data preparation | 7 | 235 |
| Outcome | 8a | 177 |
|  | 8b | Not applicable |
|  | 8c | 237 |
| Predictors | 9a | 186,190, 196 |
|  | 9b | 193,195, 202 Supplementary Table 1 and 2. |
|  | 9c | Not applicable |
| Sample size | 10 | 177, 257 |
| Missing data | 11 | Not applicable |
| Analythical methods | 12a | 236, 242 |
|  | 12b | Not applicable |
|  | 12c | 224, 230 |
|  | 12d | 242 |
|  | 12e | 233 |
|  | 12f | 229 |
|  | 12g | Not applicable |
| Class imbalance | 13 | 237 |
| Fairness | 14 | Not applicable |
| Model output | 15 | 243 |
| Training versus evaluation | 16 | 237 |
| Ethical approval | 17 | 252 |
|  | 18a | 432 |
|  | 18b | 448 |
|  | 18c | 160 |
|  | 18d | 160 |
|  | 18e | 440 |
|  | 18f | 443 |
| Patient and public involvement | 19 | Not applicable |
| Participants | 20a | 259 |
|  | 20b | Table 1 |
|  | 20c | 284, Table 2 |
| Model development | 21 | 266 |
| Model  specification | 22 | Figure 1 and 2 |
| Model performance | 23a | 269 |
|  | 23b | 269 |
| Model updating | 24 | Not applicable |
| Interpretation | 25 | 302 |
| Limitations | 26 | 387 |
| Usability of the model in the context of current  care | 27a | 389 |
|  | 27b | 400 |
|  | 27c | 407 |

Supplementary Table 3. Tripod AI checklist
